# Early recurrence prediction after curative-intent surgery of intrahepatic cholangiocarcinoma using a novel weighted tumor burden score

**DOI:** 10.1186/s12957-026-04560-8

**Published:** 2026-08-26

**Authors:** Anna Mantas, Smiths S. Lueong, Sophia Mauerer, Antonia Krumm, Dieter P. Hoyer, Iakovos Amygdalos, Tom F. Ulmer, Jens Siveke, Florian W. R. Vondran, Ulf P. Neumann, Jan Bednarsch

**Affiliations:** 1 Department of Surgery and Transplantation, University Hospital Essen, Hufelandstraße 55, 45147 Essen, Germany; 2 German Cancer Consortium (DKTK), Partner Site Essen/Düsseldorf, a partnership between German Cancer Research Center (DKFZ) and University Hospital Essen, Essen, Germany; 3 Bridge Institute of Experimental Tumor Therapy (BIT) and Division of Solid Tumor Translational Oncology (DKTK), West German Cancer Center, University Hospital Essen, University of Duisburg-Essen, Essen, Germany; 4 Department of Surgery and Transplantation, University Hospital RWTH Aachen, Aachen, Germany; 5 Department of Surgery, Maastricht University Medical Center (MUMC), Maastricht, Netherlands

**Keywords:** Early Recurrence, Intrahepatic Cholangiocarcinoma (ICC), Tumor Burden Score

## Abstract

**Background:**

Recurrence after curative-intent resection of patients with intrahepatic cholangiocarcinoma (ICC) remains common and difficult to predict. Tumor burden scores (TBS) incorporating tumor size and tumor nodule number have been proposed, but the relative contribution of multifocality remains unclear.

**Methods:**

Patients undergoing curative-intent surgery for ICC in a large hepatobiliary center between 2009 and 2024 were retrospectively analyzed. A simplified weighted tumor burden score (wTBS) was developed using tumor size and tumor nodule number (wTBS = largest tumor diameter (cm) + 5 × tumor nodule number). The score was derived in a primary cohort and externally validated in an independent cohort. Early recurrence was defined as recurrence within 12 months after surgery. Predictive performance was assessed using logistic regression analysis, receiver operating characteristic (ROC) analysis, Kaplan–Meier survival curve analysis and Cox regression models.

**Results:**

A total of 198 patients were included in this study. In the study cohort, the wTBS was significantly associated with early recurrence (OR 1.12 per point, 95% CI 1.07–1.18, *p* < 0.001) and recurrence-free survival (RFS; HR 1.06 per point, 95% CI 1.04–1.09, *p* < 0.001). ROC analysis demonstrated improved discriminatory performance compared to tumor size or tumor nodule number alone (area under the curve [AUC] 0.69). Using a cutoff of ≥ 14, patients with high wTBS had significantly shorter RFS (median 9 vs. 21 months, *p* < 0.001). These findings were confirmed in an independent validation cohort comprising 162 patients, in which wTBS remained significantly associated with early recurrence (OR 1.12) and RFS (HR 1.08, *p* < 0.001) with a median RFS of 7 vs. 26 months for high- vs. low-risk patients.

**Conclusions:**

A simplified wTBS incorporating tumor size and tumor nodule number was associated with early recurrence after ICC resection and showed consistent prognostic value in an independent cohort. Given its moderate discriminatory ability, the score should currently be regarded as an adjunct for postoperative risk stratification rather than a stand-alone tool for treatment selection. Prospective validation and evaluation of clinical utility are required before implementation in treatment algorithms.

**Supplementary Information:**

The online version contains supplementary material available at 10.1186/s12957-026-04560-8.

## Introduction

Intrahepatic cholangiocarcinoma (ICC) represents the second most common primary liver malignancy and is characterized by an aggressive tumor biology and poor prognosis. Despite advances in surgical techniques, curative-intent surgery remains the only curative treatment option. However, long-term outcomes remain limited due to factors such as high rates of tumor recurrence. Notably, recurrence occurs in up to 50–70% of patients, with the majority of cases arising within the first 12 months following surgery [1].

Accurate risk stratification of patients undergoing surgery for ICC is therefore essential for postoperative management and for identifying individuals at high risk for early recurrence. Traditional staging systems, such as the American Joint Committee on Cancer (AJCC) classification, incorporate tumor size, multifocality and vascular invasion. Yet, their prognostic performance remains suboptimal. In particular, tumor size and tumor nodule number are typically assessed as independent variables despite increasing evidence suggesting that both parameters interact to reflect the overall tumor burden and biological aggressiveness of the disease [2].

To address this limitation, the tumor burden score (TBS) has been introduced as a composite metric integrating tumor size and tumor nodule number into a single continuous variable. TBS was originally developed in the context of colorectal liver metastases (CRLM) and has emerged as a prognostic tool in hepatocellular carcinoma (HCC) and more recently in ICC as well [3]. The score is calculated as the Euclidean distance from the origin of a Cartesian plane defined by tumor size and number, thereby providing a quantitative representation of tumor morphology and burden.

Emerging evidence suggests that TBS is associated with long-term oncological outcomes, including overall survival (OS) and recurrence-free survival (RFS) [3, 4]. Furthermore, recent studies have demonstrated that combining TBS with tumor-specific biomarkers, such as carbohydrate antigen 19–9 (CA19-9) or carcinoembryonic antigen (CEA), may further improve prognostic accuracy [3].

However, despite these promising findings, several limitations remain. First, currently available TBS models rely on relatively complex mathematical formulas, which may limit their applicability in routine clinical practice. Second, existing studies have primarily focused on long-term outcomes such as OS and RFS, whereas early recurrence, representing a distinct clinical entity associated with aggressive tumor biology, has been less extensively investigated. Finally, there remains a need for simplified, clinically applicable models that can reliably predict early recurrence and support perioperative decision-making.

The aim of the present study was to develop and externally validate a simplified wTBS for the prediction of early recurrence following curative-intent surgery for ICC and to evaluate its potential as an adjunct for postoperative risk stratification that may facilitate decision-making in daily practice.

## Material and methods

### Patients

This retrospective study included all consecutive patients who underwent liver resection for ICC at the University Hospital Essen between 2009 and 2024. Patients were included if they had histologically confirmed ICC and underwent curative resection. Exclusion criteria comprised the presence of distant metastases at the time of surgery, palliative procedures, perioperative mortality, patients lost to follow-up or missing key clinicopathological data. For external validation, an independent cohort of patients undergoing curative-intent resection for histologically confirmed ICC at University Hospital RWTH Aachen between 2009 and December 2022 was analyzed. The same inclusion and exclusion criteria as in the derivation cohort were applied. Perioperative evaluation and treatment principles were comparable to those used in the derivation cohort. The study was conducted in accordance with the requirements of the local ethics committee (24–12198-BO) and followed the current version of the Declaration of Helsinki and the Good Clinical Practice guidelines (ICH-GCP). Informed consent was waived due to the retrospective study design. All clinical data were identified from a prospectively maintained institutional database and were pseudonymized for analysis.

### Preoperative evaluation and diagnostic work-up

All patients received a thorough preoperative evaluation adapted to their specific clinical circumstances. Standard imaging workup included computed tomography (CT) and/or magnetic resonance imaging (MRI) in every case. Treatment planning for all patients was conducted through a multidisciplinary tumor board following current institutional protocols. A senior hepatobiliary surgeon ultimately made the final decision to proceed with surgery and the choice of operative technique. Baseline functional status was documented according to the American Society of Anesthesiologists (ASA) status classification.

### Data collection and follow-up

Clinical, laboratory, surgical and pathological data were extracted from electronic medical records. Variables included demographic characteristics (age, sex, body mass index (BMI)), perioperative parameters (operative time, intensive care unit [ICU] stay, length of hospitalization) and laboratory values (hemoglobin, platelet count, international normalized ratio [INR], bilirubin, liver enzymes, C-reactive protein (CRP), CA19-9). Tumor-specific variables included tumor size (maximum diameter in centimeter), tumor nodule number and pathological characteristics such as T stage, nodal status, resection margin status and the presence of macrovascular invasion. Tumor size, defined as the maximum diameter of the largest lesion, and tumor nodule number were assessed exclusively on preoperative CT and/or MRI. Each patient received a regular follow-up consisting of clinical examination, standard laboratory blood tests with tumor markers and cross-sectional imaging. Additional imaging and/or biopsy was performed if tumor recurrence was suspected. By contacting either the patients themselves, the primary care physicians or the oncologists, we were able to determine a complete follow-up.

### Definition of early recurrence

Early recurrence was defined as tumor recurrence occurring within 12 months following surgical resection. Patients were categorized into two groups: Early recurrence (< 12 months), no early recurrence (recurrence ≥ 12 months postoperatively or no recurrence during follow-up).

### Definition of tumor burden scores

The conventional TBS integrates tumor size and tumor nodule number into a continuous variable.

$$TBS=\sqrt{{\left(tumor size\right)}^{2}+ {\left(tumor number\right)}^{2}}$$

In the present study, a simplified weighted TBS (wTBS) was developed to improve clinical applicability. The score was defined as:

*wTBS=tumor size+5× tumor nodule number*

The weighting factor for tumor nodule number was derived from regression coefficients (0.615 vs. 0.103) of multivariable logistic regression analysis for early recurrence, corresponding to an approximate sixfold stronger effect compared to tumor size. For improved clinical applicability, this ratio was simplified, resulting in the final score. Alternative scores using weighting factors of 4, 5 and 6 were evaluated by ROC analysis.

### Statistical analysis

RFS was defined as the period from the date of liver resection until the date of first recurrence. Patients without tumor recurrence were censored at the last follow-up or at the time of death. Patients who displayed perioperative mortality (Clavien-Dindo grade V) were excluded from analyses related to recurrence, as these patients were not at risk for the primary endpoint. Continuous variables are expressed as median with interquartile range (IQR) and compared using the Student’s t-test or Mann–Whitney U test. Categorical variables are expressed as absolute numbers and percentages and are compared using the chi-square test or Fisher’s exact test. For a detailed description of the study cohort, descriptive statistical analyses were performed. Discriminatory performance of the TBS was assessed using receiver operating characteristic (ROC) curve analysis and the area under the curve (AUC) was calculated. In the external validation cohort, model discrimination was assessed using the AUC with 95% confidence interval. Sensitivity and specificity were calculated using the predefined wTBS cutoff of ≥ 14. Model calibration was evaluated using calibration plots, the calibration intercept, calibration slope, and Brier score. Decision curve analysis was performed to assess net benefit across a range of threshold probabilities.

Univariable logistic regression analysis was performed to identify factors associated with early recurrence. Variables with a *p*-value < 0.05 in univariable analysis were included in multivariable logistic regression models to identify independent predictors. Results are reported as odds ratios (OR) with 95% confidence intervals (CI). RFS was analyzed using the Kaplan–Meier method and compared using the log-rank test. Cox proportional hazards regression analysis was performed to identify factors associated with RFS with results reported as hazard ratios (HR) and 95% CI. The number of events per variable (EPV) was calculated for both Cox and logistic regression models to assess model stability. The proportional hazards assumption was assessed using Schoenfeld residuals. A two-sided p-value < 0.05 was considered statistically significant. Statistical analyses were performed using SPSS software (SPSS Statistics 24, IBM Corp., Armonk, NY, USA) and R software (R Foundation for Statistical Computing, Vienna, Austria).

## Results

### Patient cohort

A total of 208 patients underwent curative-intent liver surgery for ICC at the institution. After application of the exclusion criteria, 198 patients were eligible for analysis (2 patients were lost to follow-up, 8 patients displayed perioperative mortality). Early recurrence within 12 months occurred in 93 patients (47%). Patients with early recurrence presented with significantly larger tumors (8 cm vs 6 cm, *p* = 0.003) and a higher number of tumor nodules (*p* < 0.001, Table 1), resulting in higher tumor burden, as reflected by significantly higher tumor burden scores (8.4 vs 6.7, *p* = 0.001). Preoperative systemic therapy was administered to 18 patients (8.7%), postoperative systemic therapy to 119 patients (60.1%). This consisted predominantly of chemotherapy, which was administered to 118 patients (59.6%), while postoperative radiotherapy was documented in 15 of 196 patients with available data (6.6%). R1 resection was documented in 54 patients (26.0%). Baseline demographic and laboratory parameters did not differ significantly between the early- and no-early-recurrence groups except a longer operative time in the early recurrence group (*p* = 0.008, Table 1).

**Table 1:** Study cohort

Variables	ICC cohort (n = 208)	Early Recurrence (n = 93)	No early recurrence (n = 105)	*p*-value
**Demographics**				
Sex, m/f (%)	96 (46)/112 (54)	40 (43)/53 (57)	50 (48)/55 (52)	
Age (years)	66 (57–72)	64 (54–71)	66 (59–73)	
BMI (kg/m2)	25.6 (23–29)	25.3 (23–28)	26.5 (22–30)	
Preoperative systemic chemotherapy, n (%)	18 (8.7)	12 (12.9)	5 (4.8)	
Time to surgery (days)	40 (22–84)	38 (21–89)	40 (23–86)	
ASA, n (%)				
I	2 (1)	1 (1.1)	1 (1)	
II	95 (46)	49 (53)	44 (42)	
III	107 (51)	43 (46)	59 (56)	
IV	4 (2)	0	1 (1)	
V	0	0	0	
**Preoperative laboratory parameters**				
Hemoglobin (g/dL)	13.3 (12–14)	13.3 (12–14)	13.3 (12–14)	
Platelet count (/nL)	248 (191–308)	258 (189–321)	244 (196–301)	
Prothrombin time (%)	102 (92–111)	103 (94–113)	98 (87–110)	
INR	0.99 (0.95–1.05)	0.99 (0.95–1.03)	1.01 (0.96–1.06)	
AST (U/L)	32 (24–42)	31 (23–46)	32 (23–40)	
ALT (U/L)	29 (21–42)	27 (21–42)	30 (22–40)	
Total bilirubin (mg/dL)	0.5 (0.4–0.7)	0.6 (0.4–0.7)	0.5 (0.4–0.7)	
gGT (U/L)	91 (49–211)	105 (56–218)	78 (44–197)	
CRP (mg/dL)	0.6 (0.4–1.7)	0.6 (0.4–1.7)	0.5 (0.4–1.2)	
CA19-9 (U/mL)	43 (13–321)	47 (11–221)	30 (12–246)	
**Tumor characteristics**				
Tumor size (cm)	7 (4.3–9.4)	8 (4.9–10.3)	6 (3.8–8.5)	0.003
Tumor nodule number, n (%)				< 0.001
1	138 (66.7)	50 (53.8)	83 (79)	
2–3	40 (19.3)	21 (22.6)	17 (16.2)	
4–5	4 (1.9)	3 (3.2)	1 (1)	
> 5	25 (12.1)	19 (20.4)	4 (3.9)	
TBS	7 (4.6–9.6)	8 (5–10.8)	6 (3.9–8.6)	0.001
Simplified wTBS	13.5 (10–18.5)	16.4 (11.3–22.5)	12 (9.3–15.4)	
N1, n (%)	53 (46.9)	36 (58.1)	15 (33.3)	
R1 resection, n (%)	54 (26)	24 (25.8)	27 (25.8)	
Grading, n (%)				
G1	4 (1.9)	0	4 (3.8)	
G2	148 (71.8)	61 (67)	78 (74.3)	
G3	54 (26.3)	30 (33)	23 (21.9)	
G4	0	0	0	
MVI, n (%)	48 (24.6)	24 (27.5)	21 (21.4)	
LVI, n (%)	37 (18.7)	22 (24.4)	14 (14.3)	
pT category, n (%)				
1	101 (48.6)	33 (35.5)	63 (60)	
2	90 (43.3)	48 (51.6)	37 (35.2)	
3	9 (4.4)	6 (6.5)	3 (2.9)	
4	8 (3.8)	6 (6.5)	2 (1.9)	
**Operative data**				
Operative time (minutes)	175 (136–231)	190 (145–233)	163 (127–230)	0.008
Intraoperative blood transfusion, n (%)	23 (11.1)	9 (9.7)	12 (11.4)	
**Postoperative data**				
Intensive care stay (days)	1 (1 −3)	1 (1–2)	1 (1–3)	
Hospitalization (days)	11 (8–19)	11 (8–19)	12 (8–18)	
Postoperative complications, n (%)				
No complications	104 (50)	50 (53.8)	53 (50.5)	
Clavien-Dindo I	20 (9.6)	9 (9.7)	11 (10.5)	
Clavien-Dindo II	30 (14.4)	15 (16.1)	15 (14.3)	
Clavien-Dindo IIIa	20 (9.6)	10 (10.8)	10 (9.5)	
Clavien-Dindo IIIb	10 (4.8)	5 (5.4)	5 (4.8)	
Clavien-Dindo IVa	13 (6.3)	4 (4.3)	9 (8.6)	
Clavien-Dindo IVb	3 (1.4)	0	2 (1.9)	
Clavien-Dindo V	8 (3.8)	0*	0*	
Postoperative systemic therapy	119 (60.1)	61 (65.6)	58 (55.2)	
Chemotherapy	118 (59.6)	61 (65.6)	57 (54.3)	
Radiotherapy	15 (6.6)	6 (6.5)	7 (6.7)	
Median RFS, months (95% CI)	11 (5–31)	6 (3–8)	30 (15–56)	

Data presented as median and interquartile range if not noted otherwise. Early recurrence status was unavailable for 10 patients

ALT, alanine aminotransferase; ASA, American Society of Anesthesiologists classification; AST, aspartate aminotransferase; BMI, body mass index; CA19-9, carbohydrate antigen 19–9; CRP, C-reactive protein; gGT, gamma glutamyl transferase; INR, international normalized ratio; LVI, lympho-vascular invasion; MVI, microvascular invasion; RFS, recurrence-free survival; TBS, tumor burden score; wTBS, weighted tumor burden score

### Creation of simplified tumor score

**Fig. 1:**
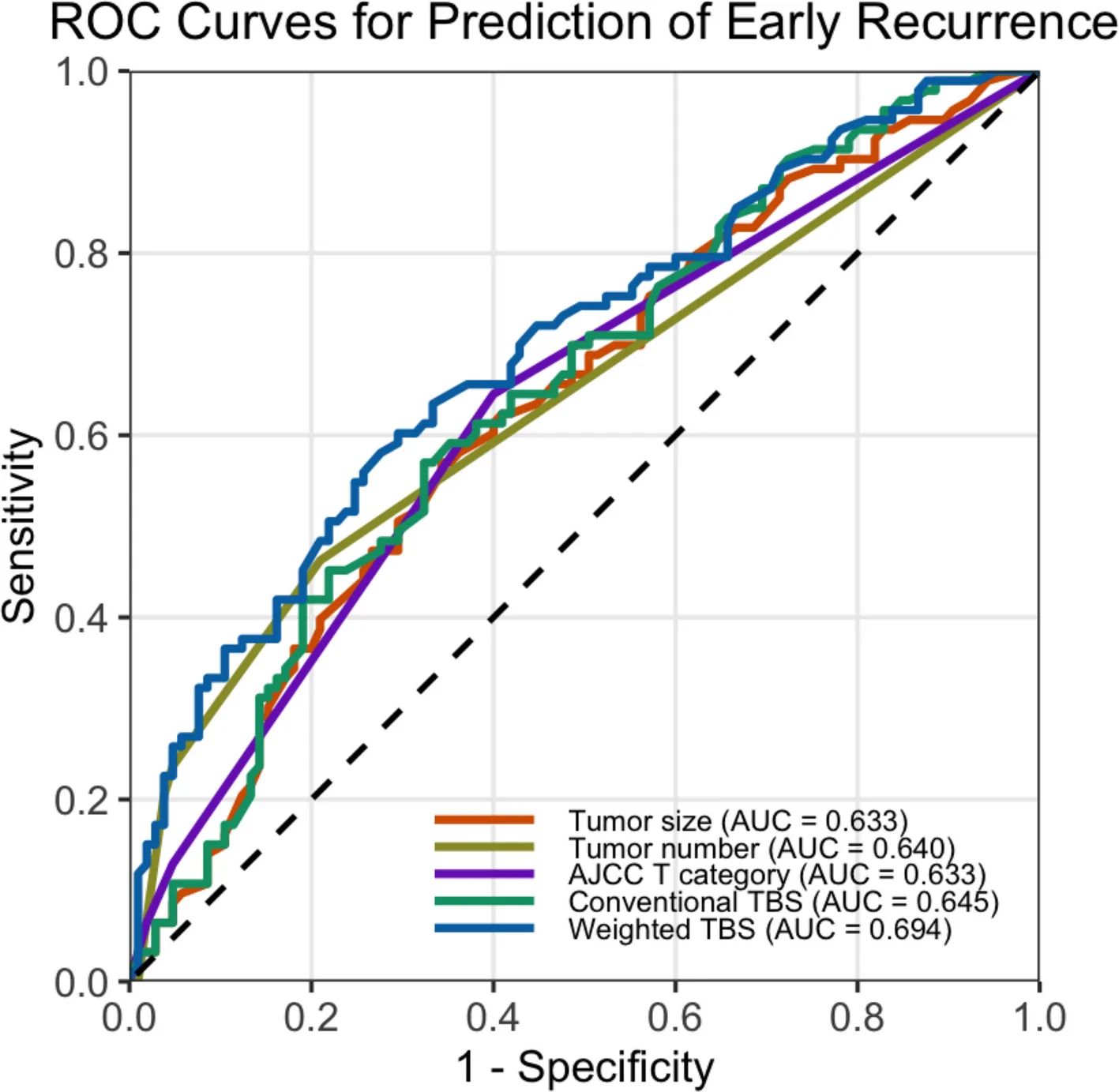
ROC curve analysis for prediction of early recurrence. The simplified wTBS showed the highest AUC (0.694), followed by conventional TBS (0.645), tumor nodule number (0.640), tumor size (0.633) and AJCC T category (0.633). The dashed line represents the line of no discrimination

To identify the effect of tumor size and nodule number on early recurrence, logistic regression analysis was performed. Both variables were independently associated with early recurrence. Tumor size was associated with a moderate increase in risk (OR 1.11 per cm, 95% CI 1.03–1.20, *p* = 0.01), whereas tumor nodule number showed a markedly stronger effect with each additional tumor nodule nearly doubling the risk (OR 1.85, 95% CI 1.32–2.59, *p* < 0.001, Table 2A). Based on the regression coefficient ratio of both variables, a weighted TBS was simplified to a weighting factor of 5 for improved clinical applicability. Alternative integer weightings yielded nearly identical discriminatory performance. The AUCs were 0.691 (95% CI 0.618–0.765) for factor 4, 0.695 (95% CI 0.622–0.768) for factor 5 and 0.694 (95% CI 0.621–0.767) for factor 6. ROC curve analysis demonstrated that the simplified weighted TBS (wTBS) showed improved discriminatory performance compared to tumor size, tumor nodule number alone, conventional TBS as well as AJCC T category. The AUC for wTBS was higher (0.694) than for tumor size (0.633), tumor nodule number (0.640), conventional TBS (0.645) and AJCC T category (0.633), indicating the highest numerical AUC among the evaluated measures for early recurrence (Fig. 1). Compared with the conventional TBS (AUC 0.645, 95% CI 0.569–0.722) and the AJCC T category (AUC 0.633, 95% CI 0.556–0.711), the weighted TBS demonstrated improved discrimination for early recurrence (AUC 0.694, 95% CI 0.621–0.767). Paired ROC analysis demonstrated that the wTBS had the highest discriminatory performance for early recurrence (AUC 0.694, 95% CI 0.621–0.767).

**Table 2:** Derivation of the weighted tumor burden score and logistic regression analysis for early recurrence

A. Derivation model for the weighted score					
**Variable**	**ß regression coefficients**		**OR (95% CI)**	***p*-value**	
Tumor size (per cm)	.103		1.11 (1.03–1.20)	0.01	
Tumor nodule number	.615		1.85 (1.32–2.59)	< 0.001	

**B. Univariable and multivariable logistic regression for early recurrence**					
	**Univariate analysis**		**Multivariate analysis**		
	**OR (95% CI)**	***p*-value**	**OR (95% CI)**		***p*-value**
**Demographics**					
Sex (male = 1)	1.15 (0.66–2.02)	0.619			
Age (≤ 65 years = 1)	0.72 (0.41–1.26)	0.252			
BMI (≤ 25 kg/m^2^ = 1)	0.73 (0.42–1.30)	0.283			
Preoperative systemic chemotherapy (no = 1)	**2.96 (1.00–8.76)**	**0.049**	1.74 (0.36–8.5)		0.491
Time to surgery (≤ 60 days = 1)	0.79 (0.44–1.40)	0.417			
ASA (I/II = 1)	0.65 (0.37–1.13)	0.126			
**Preoperative laboratory parameters**					
Hemoglobin (≤ 13 g/dL = 1)	0.98 (0.56–1.72)	0.955			
Platelet count (≤ 300/nL = 1)	1.38 (0.74–2.55)	0.311			
Prothrombin time (≤ 100% = 1)	**1.92 (1.08–3.40)**	**0.026**	1.29 (0.49–3.43)		0.608
INR (≤ 1 = 1)	0.63 (0.36–1.11)	0.111			
AST (≤ 40 U/L = 1)	1.53 (0.82–2.86)	0.184			
ALT (≤ 40 U/L = 1)	1.09 (0.57–2.08)	0.797			
Bilirubin (≤ 1 mg/dL = 1)	1.76 (0.71–4.32)	0.221			
gGT (≤ 90 U/L = 1)	1.53 (0.87–2.68)	0.141			
CRP (≤ 1 mg/dL = 1)	1.49 (0.79–2.83)	0.218			
CA19-9 (≤ 100 U/mL = 1)	1.20 (0.63–2.31)	0.580			
**Tumor characteristics**					
Simplified wTBS	**1.12 (1.07–1.18)**	** <.001**	**1.16 (1.07–1.26)**		** < 0.001**
N1 (no = 1)	**2.80 (1.25–6.26)**	**0.012**	excl.*		
R1 resection (no = 1)	0.89 (0.12–6.45)	0.908			
Grading (G1/G2 = 1)	1.64 (0.86–3.11)	0.134			
MVI (no = 1)	1.36 (0.69–2.68)	0.375			
LVI (no = 1)	**2.47 (1.15–5.32)**	**0.021**	0.43 (0.13–1.46)		0.176
pT category (T1/T2 = 1)	**2.28 (1.05–4.94)**	**0.038**	1.3 (0.39–4.27)		0.671

Data are presented as odds ratios (ORs) with 95% confidence intervals (CIs). The weighted tumor burden score was derived from the β regression coefficients of tumor size and tumor nodule number. ALT, alanine aminotransferase; ASA, American Society of Anesthesiologists; AST, aspartate aminotransferase; BMI, body mass index; CA19-9, carbohydrate antigen 19–9; CRP, C-reactive protein; gGT, gamma-glutamyl transferase; INR, international normalized ratio; LVI, lymphovascular invasion; MVI, microvascular invasion; OR, odds ratio; wTBS, weighted tumor burden score. *Excluded from the multivariable model

Differences compared with tumor nodule number (0.640, *p* = 0.072) and AJCC T category (0.633, *p* = 0.124) were not statistically significant. With the help of Youden-Index, wTBS was dichotomized into high- and low-risk groups. An optimal cut-off value of ≥ 14 was identified, yielding a sensitivity of 61% and a specificity of 68%.

### Univariable and multivariable logistic regression analysis for early recurrence

Univariable logistic regression analysis was performed to identify factors associated with early recurrence. The simplified wTBS was significantly associated with early recurrence (OR 1.12, 95% CI 1.07–1.18, *p* < 0.001). Among clinicopathological variables, nodal involvement was significantly associated with early recurrence (*p* = 0.012) as well as lymphovascular invasion (*p* = 0.021) and advanced tumor stage (*p* = 0.038). Regarding treatment-related factors, patients who received neoadjuvant chemotherapy had higher odds of early recurrence (OR 2.96, 95% CI 1–8.76, *p* = 0.049). Similarly, coagulation reflected by preoperative prothrombin value was significantly associated with early recurrence (OR 1.92, 95% CI 1.08–3.40, *p* = 0.026). In contrast, several demographic and laboratory parameters were not significantly associated with early recurrence, including age, sex, BMI, ASA status, preoperative hemoglobin and platelet count, total bilirubin, ALT, gGT, CRP and CA19-9 (Table 2B).

Variables with a *p*-value < 0.05 in univariable analysis were included in the multivariable logistic regression model. Nodal status was excluded due to missing values in 91 patients. In multivariable logistic regression analysis, the simplified wTBS remained an independent predictor of early recurrence (OR 1.16, 95% CI 1.07–1.26, *p* < 0.001, Table 2). In a complete-case sensitivity analysis restricted to patients with documented nodal status (*n* = 107), wTBS remained independently associated with early recurrence after adjustment for nodal positivity (OR 1.12 per point, 95% CI 1.04–1.19, *p* = 0.002). Nodal positivity was also independently associated with early recurrence (OR 3.58, 95% CI 1.50–8.57, *p* = 0.004). In the final multivariable logistic regression analysis, 88 events were available for 5 variables, resulting in an EPV of 17.6 indicating model stability. Leave-one-covariate-out sensitivity analyses were performed and yielded ORs ranging from 1.11 to 1.13 for wTBS and statistical significance in all reduced models indicating model robustness (Supplementary Table 2 A).

### Univariable and multivariable Cox regression analysis for recurrence-free survival

In univariable Cox regression analysis, the simplified wTBS was significantly associated with RFS (Table 3). Each one-point increase in the score was associated with a 6.1% increase in the hazard of recurrence (HR 1.06, 95% CI 1.04–1.09, *p* < 0.001). Among clinicopathological variables, nodal positivity (HR 2.01, 95% CI 1.31–3.07, *p* = 0.001), advanced tumor stage (HR 2.20, 95% CI 1.30–3.71, *p* = 0.003) and several histopathological findings were significantly associated with RFS. Variables with a p-value < 0.05 in univariable analysis were included in the multivariable Cox regression model. Due to missing nodal status and CA19-9 values, a total of 182 cases were included in the multivariable model. When transferred onto the multivariable Cox regression model, wTBS remained independently associated with RFS (HR 1.06, 95% CI 1.03–1.09, *p* < 0.001) as well as tumor grading (HR 1.66, 95% CI 1.14–2.41, *p* = 0.008). In the final multivariable Cox regression model, 137 events were available for 5 variables, resulting in an EPV of 27.4 indicating model stability (Supplementary Table 1). Leave-one-covariate-out sensitivity analyses confirmed the stability of this association with stable HRs for wTBS ranging from 1.06 to 1.07 after sequential removal of individual covariates from the final model, all *p* < 0.001 (Supplementary Table 2B). Further, Schoenfeld residuals indicated no significant violation of the proportional hazards assumption for any covariate or globally (global *p* = 0.42, Supplementary Fig. 1).

**Table 3:** Cox regression analysis for RFS

	**Univariate analysis**		**Multivariate analysis**	
	**HR (95% CI)**	***p*-value**	**HR (95% CI)**	***p*-value**
**Demographics**				
Sex (female = 1)	0.99 (0.72–1.37)	0.956		
Age (> 65 years = 1)	1.01 (0.73–1.40)	0.943		
BMI (> 25 kg/m^2^ = 1)	0.88 (0.64–1.23)	0.462		
Preoperative systemic chemotherapy (yes = 1)	1.69 (0.97–2.94)	0.064		
Time to surgery (> 60 days = 1)	0.86 (0.61–1.19)	0.365		
ASA (III/IV = 1)	0.85 (0.61–1.17)	0.306		
**Preoperative laboratory parameters**				
Hemoglobin (> 13 g/dL = 1)	0.94 (0.68–1.29)	0.691		
Platelet count (> 300/nL = 1)	1.26 (0.64–2.48)	0.501		
Prothrombin time (< 100% = 1)	**1.45 (1.04–2.03)**	**0.028**	1.17 (0.83–1.65)	0.380
INR (> 1 = 1)	0.77 (0.55–1.06)	0.112		
AST (> 40 U/L = 1)	1.20 (0.84–1.72)	0.321		
ALT (> 40 U/L = 1)	0.97 (0.66–1.41)	0.856		
Bilirubin (> 1 mg/dL = 1)	1.40 (0.87–2.25)	0.164		
gGT (> 90 U/L = 1)	1.24 (0.88–1.75)	0.217		
CRP (> 1 mg/dL = 1)	1.31 (0.90–1.90)	0.154		
CA19-9 (> 100 U/mL = 1)	**1.54 (1.07–2.23)**	**0.021**	excl.*	
**Tumor characteristics**				
Simplified wTBS	**1.06 (1.04–1.09)**	** < 0.001**	**1.06 (1.03–1.09)**	** < 0.001**
N1 (no = 1)	**2.01 (1.31–3.07)**	**0.001**	excl.*	
R1 resection (no = 1)	1.20 (0.83–1.73)	0.322		
Grading (G1/G2 = 1)	**1.56 (1.08–2.25)**	**0.017**	**1.66 (1.14–2.41)**	**0.008**
MVI ( no = 1)	1.39 (0.95–2.03)	0.094		
LVI ( no = 1)	**1.86 (1.25–2.77)**	**0.002**	1.47 (0.97–2.23)	0.068
pT category (T1/T2 = 1)	**2.20 (1.30–3.71)**	**0.003**	1.33 (0.70–2.52)	0.385

Data are presented as hazard ratios (HRs) with 95% confidence intervals (CIs). ALT, alanine aminotransferase; ASA, American Society of Anesthesiologists; AST, aspartate aminotransferase; BMI, body mass index; CA19-9, carbohydrate antigen 19–9; CRP, C-reactive protein; gGT, gamma-glutamyl transferase; INR, international normalized ratio; LVI, lymphovascular invasion; MVI, microvascular invasion; RFS, recurrence-free survival; wTBS, weighted tumor burden score. *Excluded from the multivariable model

### Survival analysis

**Fig. 2:**
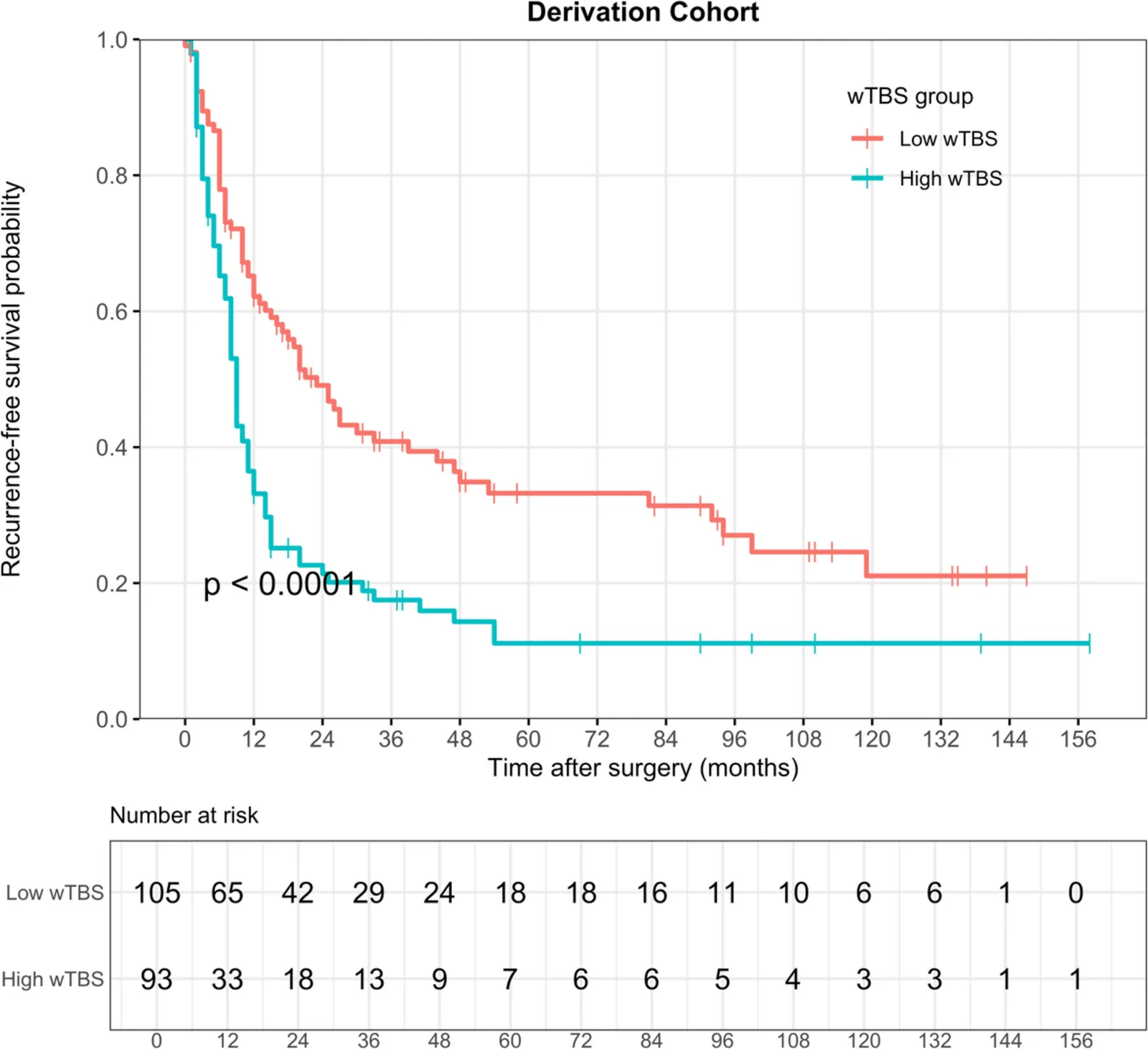
RFS according to wTBS in the study cohort. Kaplan–Meier analysis for RFS stratified by simplified wTBS. Patients with a score ≥ 14 showed significantly shorter RFS compared to those with a score < 14 (9 vs. 21 months, log-rank *p* < 0.001)

Kaplan–Meier analysis demonstrated a significantly reduced RFS in patients with higher wTBS values (Fig. 2). The median RFS was 9 months (95% CI 7.9–10.1) in the high-risk group vs. 21 months (95% CI 13.9–28.1) in the low-risk group. This difference was statistically significant (log-rank *p* < 0.001), indicating a clear separation of survival curves according to risk stratification by the simplified wTBS.

### External validation

**Fig. 3:**
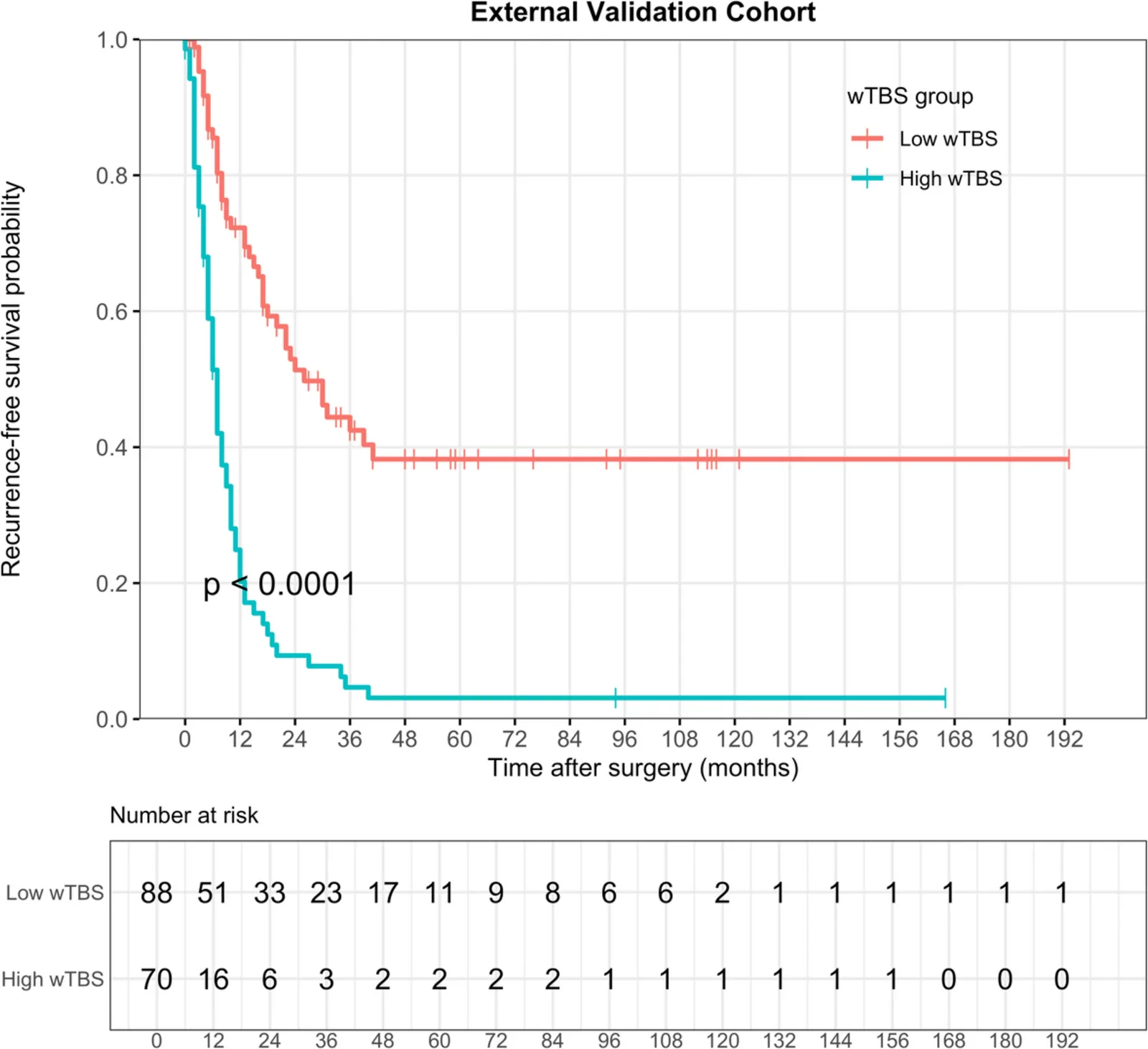
RFS according to wTBS in the validation cohort. Kaplan–Meier analysis of RFS stratified by the simplified wTBS (cut-off ≥ 14) in the external validation cohort of patients undergoing curative resection. Patients with high-risk wTBS demonstrated significantly worse RFS compared to low-risk patients. Median RFS was 7 months in the high-risk group vs. 26 months in the low-risk group (log-rank *p* < 0.001)

The prognostic value of the simplified wTBS was confirmed in an independent external validation cohort (*n* = 162). The median follow-up was 20 months (IQR 10–48 months). Relevant details of the cohort are shown in Supplementary Table 3. In this cohort, wTBS remained significantly associated with RFS (7 vs. 26 months, log-rank *p* < 0.001) and demonstrated comparable predictive performance, supporting the robustness and generalizability of the model (Fig. 3). Calibration analysis demonstrated good agreement between predicted and observed probabilities in both the derivation and external validation cohorts. In the external validation cohort, the wTBS showed a calibration intercept of − 0.057 and a calibration slope of 1.053 with a Brier score of 0.21. Decision curve analysis suggested potential net benefit across a range of threshold probabilities (Supplementary Figs. 2–5, Supplementary Table 4).

## Discussion and conclusion

The present study demonstrates that a simplified weighted TBS, integrating tumor size and tumor nodule number with an emphasis on multifocality, is significantly associated with early recurrence (defined as recurrence within 12 months postoperatively) following curative-intent resection for ICC (OR 1.16, 95% CI 1.07–1.26, *p* < 0.001), while in Cox regression it was independently associated with RFS (HR 1.06, 95% CI 1.03–1.09, *p* < 0.001). WTBS remained an independent predictor in multivariable analysis and showed consistent prognostic performance in an external validation cohort, thereby underlining its robustness and potential clinical applicability.

These findings align with previous studies highlighting the prognostic relevance of tumor burden, incorporating both tumor size and tumor nodule number, in hepatobiliary malignancies. The concept of the TBS, initially introduced in CRLM by Sasaki et al. and later validated in HCC and ICC, integrates tumor size and number into a continuous variable and has been associated with survival outcomes [5]. However, most existing models focus primarily on long-term endpoints such as OS and RFS, while early recurrence, representing a surrogate of aggressive tumor biology, has been less extensively investigated [6]. Our data extend the TBS concept from a general survival marker to a pragmatic tool for identifying patients at risk of biologically aggressive, early postoperative recurrence.

Recent studies have applied more complex prediction approaches to different clinical endpoints in ICC. Huang et al. developed an interpretable machine-learning model for the preoperative prediction of textbook outcome, emphasizing the importance of model transparency, external validation, and clinical decision support [7]. In contrast, the present wTBS was intentionally designed as a simple morphologic score for the prediction of early recurrence. Furthermore, Tsilimigras et al. developed an international 10-point score for post-recurrence survival, underscoring that risk stratification remains relevant not only before recurrence but also for subsequent treatment selection and prognosis [8]. Together, these studies illustrate the complementary roles of simple clinically applicable scores and more complex multivariable prediction models across different stages of ICC care.

A key finding of the present study is the disproportionately strong impact of tumor multifocality on early recurrence. While tumor size showed only a moderate association, each additional tumor nodule nearly doubled the risk of early recurrence (OR 1.85 vs. OR 1.11). This observation is consistent with previous reports demonstrating that multifocal disease reflects more aggressive tumor biology, including intrahepatic dissemination or multicentric tumorigenesis [9]. In ICC, multifocality has repeatedly been identified as an independent predictor of poor oncological outcomes and is incorporated into current staging systems such as the AJCC classification [10]. Recent literature on multifocal ICC by Zhang et al. suggests that the number of lesions carries distinct prognostic information, yet their only focus remained resectability and long-term survival rather than the timing of recurrence [11]. By assigning a higher weight to tumor nodule number, the proposed wTBS captures this biological relevance more accurately than conventional approaches treating both variables equally. Early recurrence is clinically of utmost importance due to several factors besides the likely aggressive tumor growth pattern. Early recurrence, defined as recurrence within 12 months postoperatively, is frequent. In our large European cohort, 47% of patients developed such. Previous studies on ICC have shown similar results [12, 13]. Moreover, early recurrence is associated with poor oncological survival, supporting the interpretation of early recurrence being a meaningful clinical endpoint rather than a simple time-based subgroup. Kojima et al. already demonstrated in 2020 median OS of 39.5 months after re-resection of recurrent ICC versus 14.3 months after palliative treatment and 3 months after best supportive care. When recurrence occurs, therapies differ. Selected patients may undergo repeat surgery or local ablative treatment, whereas others require locoregional or systemic therapy [14–16].

In ROC analysis, the simplified wTBS demonstrated moderate but improved discriminatory performance (AUC 0.69) compared to tumor size (AUC 0.63) and tumor nodule number (AUC 0.64). Despite its improvement, the overall discriminatory performance remained moderate, which is comparable to previously published prognostic models in ICC [17]. This reflects the complex and multifactorial nature of tumor recurrence, which is influenced not only by tumor morphology but also by biological factors such as tumor differentiation, vascular invasion and molecular characteristics [1, 18]. Nevertheless, the identified cutoff (≥ 14) allowed for clinically meaningful risk stratification with significantly different RFS between low- and high-risk groups in both cohorts.

From a clinical perspective, the simplicity of the wTBS represents a major strength. Patients with a wTBS ≥ 14 showed significantly reduced RFS compared to those with lower scores (median RFS 9 vs. 21 months, *p* < 0.001). Unlike traditional TBS models requiring more complex mathematical calculations, the proposed score can be easily derived from routinely available preoperative imaging data. This facilitates its implementation in daily clinical practice and multidisciplinary decision-making. Adjuvant therapy has become more relevant in biliary tract cancer over the recent years, particularly after the BILCAP trial established capecitabine as a standard postoperative option for resected disease [19]. Since survival after curative-intent treatment of recurrent ICC is superior to that achieved with non-surgical or purely palliative approaches, a simple preoperative marker identifying patients at high risk of early recurrence may be clinically valuable for refining postoperative surveillance intensity. However, the present study does not provide evidence that wTBS-guided surveillance, adjuvant treatment or neoadjuvant strategies improve patient outcomes. The proposed wTBS is primarily based on routinely available preoperative imaging variables and is therefore straightforward to calculate. In this context, the main strength of the proposed wTBS is that it relies exclusively on preoperative imaging-derived variables, is therefore non-invasive and easy to assess. This observation may also be viewed in the context of previous work from our group showing that time to surgery was not an independent oncological risk factor in patients undergoing curative-intent resection for ICC [20]. Taken together, these findings suggest that not all readily available preoperative variables carry the same biological relevance. Morphologic tumor burden, reflecting both tumor size and tumor nodule number, appears to be more closely associated with early postoperative recurrence than treatment timing alone. However, the present study did not evaluate whether wTBS-guided surveillance or treatment allocation improves patient outcomes. The observed association therefore supports further prospective evaluation of wTBS as an adjunct for postoperative risk stratification, but does not justify recommendations regarding intensified surveillance, adjuvant treatment selection, or neoadjuvant therapy on the basis of the score alone.

Interestingly, neoadjuvant chemotherapy was associated with early recurrence in univariable analysis (OR 2.96, *p* = 0.049) but did not remain significant in multivariable models. This likely reflects selection bias, as patients receiving neoadjuvant treatment typically present with more advanced or biologically aggressive disease. Similar observations have been reported in previous studies where treatment-related variables act as surrogates for tumor biology rather than independent predictors [21]. Moreover, only a subset of patients (8.7%) received neoadjuvant systemic therapy in our cohort. This should be interpreted carefully as the small number of cases may limit statistical power.

Notably, the number of EPV was 27.4 in the final multivariable Cox regression model for RFS and 17.6 in the logistic regression model for early recurrence. Both values exceeded commonly recommended thresholds, indicating adequate model stability and suggesting low risk of overfitting.

Several limitations should be acknowledged. First, the retrospective design is associated with inherent selection bias and potential residual confounding. Second, although external validation was performed, both cohorts originate from high-volume centers, potentially limiting generalizability. Third, the discriminatory performance of wTBS was moderate, and its incremental value over established prognostic tools and its clinical net benefit require careful interpretation. Fourth, important biological factors such as molecular alterations or genomic profiling were not available. Previous studies have suggested that combining morphological scores with biomarkers such as CA19-9 or inflammatory parameters may further improve prognostic accuracy [22]. Fifth, the sample sizes of both the derivation and validation cohorts were moderate. Moreover, postoperative surveillance was based on institutional practice and individual clinical circumstances and was not fully standardized across patients and centers. Finally, a substantial proportion of missing data, particularly for nodal status, led to exclusion of cases in multivariable analyses, which may have led to important information loss. Although wTBS remained significant in a complete-case sensitivity analysis adjusting for nodal involvement, selection bias due to missing nodal assessment and residual confounding by nodal disease cannot be excluded.

In conclusion, our study introduces and externally validates a simplified wTBS for the prediction of early recurrence after curative-intent resection of ICC. By emphasizing the role of tumor multifocality and offering a clinically applicable tool, the wTBS may improve risk stratification and guide postoperative management. However, its moderate discrimination and the retrospective design preclude its use as a stand-alone instrument for surveillance or treatment decisions. Future prospective studies integrating morphological and biological factors are warranted to further refine prediction models.

## Supplementary Information

Supplementary Material 1.

## Data Availability

The datasets generated and/or analysed during the current study are not publicly available due to data protection regulations and institutional restrictions concerning patient-related clinical data, but are available from the corresponding author on reasonable request and subject to approval by the responsible institution.

## Abbreviations

ALT: Alanine aminotransferase
ASA: American Society of Anesthesiologists
AST: Aspartate aminotransferase
AUC: Area under the curve
BMI: Body mass index
CA19-9: Carbohydrate antigen 19–9
CEA: Carcinoembryonic antigen
CRLM: Colorectal liver metastases
CRP: C-reactive protein
CT: Computed tomography
EPV: Events per variable
GGT: Gamma glutamyl transferase
HCC: Hepatocellular carcinoma
ICC: Intrahepatic cholangiocarcinoma
ICU: Intensive care unit
IQR: Interquartile range
INR: International normalized ratio
MRI: Magnetic resonance imaging
OR: Odds ratio
OS: Overall survival
RFS: Recurrence-free survival
ROC: Receiver operating characteristic
TBS: Tumor burden score
wTBS: Weighted tumor burden score
