# Supplementary material for: Early recurrence prediction after curative-intent surgery of intrahepatic cholangiocarcinoma using a novel weighted tumor burden score
Source: World J Surg Oncol. 2026 Aug 26;24:347. doi: 10.1186/s12957-026-04560-8 (PMC13508320; doi:10.1186/s12957-026-04560-8)
Supplement: Supplementary file 1 [file 12957_2026_4560_MOESM1_ESM.docx]

**SUPPLEMENTARY MATERIAL**

**Supplementary Figure 1: Schoenfeld residual plots for the covariates included in the multivariable Cox regression model.**

For the simplified wTBS, prothrombin time, tumor grading, lymphatic invasion and tumor stage no evidence of violation of the proportional hazards assumption was observed (p = 0.42).

**Supplementary Figure 2: Calibration plot of the wTBS in the derivation cohort.**

The solid line represents the calibration curve estimated by logistic recalibration, and the shaded area indicates the 95% confidence interval. The dashed diagonal line represents perfect agreement between predicted and observed probabilities. Points correspond to deciles of predicted risk with 95% confidence intervals.

**Supplementary Figure 3: Calibration plot of the wTBS in the external validation cohort.**

The solid line represents the calibration curve estimated by logistic recalibration, and the shaded area indicates the 95% confidence interval. The dashed diagonal line represents perfect agreement between predicted and observed probabilities. Points correspond to deciles of predicted risk with 95% confidence intervals.

**Supplementary Figure 4: Decision curve analysis of the wTBS in the derivation cohort.**

The net benefit of the wTBS (solid line) is compared with the strategies of treating all patients (dashed line) or treating no patients (dotted line) across a range of threshold probabilities.

**Supplementary Figure 5: Decision curve analysis of the wTBS in the external validation cohort.**

The net benefit of the wTBS (solid line) is compared with the strategies of treating all patients (dashed line) or treating no patients (dotted line) across a range of threshold probabilities.

**Supplementary Table 1: Events per variable in multivariable regression models**

| **Model** | **Event definition** | **Events, n** | **Variables, n** | **EPV** |
| --- | --- | --- | --- | --- |
| Logistic regression | Early recurrence | 88 | 5 | 17.6 |
| Cox regression | Recurrence-free survival | 137 | 5 | 27.4 |

EPV=events per variable, calculated as number of events divided by number of covariates included in the multivariable model.

**Supplementary Table 2A: Leave-one-covariate-out sensitivity analysis of the final multivariable logistic regression model for early recurrence**

| **Model** | **Omitted covariate** | **N** | **Events** | **OR for wTBS** | **95% CI** | **p-value** |
| --- | --- | --- | --- | --- | --- | --- |
| Final multivariable model | None | 185 | 88 | 1.16 | 1.07-1.26 | <0.001 |
| Leave-one-out model | Prothrombin time | 187 | 90 | 1.13 | 1.07-1.19 | <0.001 |
| Leave-one-out model | Lymphovascular invasion | 196 | 91 | 1.13 | 1.07-1.19 | <0.001 |
| Leave-one-out model | Tumor stage | 185 | 88 | 1.13 | 1.06-1.19 | <0.001 |
| Leave-one-out model | Preoperative systemic chemotherapy | 185 | 88 | 1.13 | 1.06-1.19 | <0.001 |

wTBS was retained in all models. In each leave-one-out model within the complete-case population, one covariate from the final multivariable logistic regression model was removed sequentially. ORs refer to the effect estimate of wTBS.

**Supplementary Table 2B: Leave-one-covariate-out sensitivity analysis of the final multivariable Cox regression model for RFS**

| **Model** | **Omitted covariate** | **N** | **Events** | **HR for wTBS** | **95% CI** | **p-value** |
| --- | --- | --- | --- | --- | --- | --- |
| Final multivariable model | None | 182 | 137 | 1.06 | 1.03-1.09 | <0.001 |
| Leave-one-out model | Prothrombin time | 182 | 137 | 1.06 | 1.04-1.09 | <0.001 |
| Leave-one-out model | Lymphovascular invasion | 182 | 137 | 1.06 | 1.04-1.10 | <0.001 |
| Leave-one-out model | Tumor stage | 182 | 137 | 1.07 | 1.04-1.10 | <0.001 |
| Leave-one-out model | Tumor grading | 182 | 137 | 1.06 | 1.03-1.09 | <0.001 |

wTBS was retained in all models. In each leave-one-out model within the complete-case population, one covariate from the final multivariable Cox regression model was removed sequentially. HRs refer to the effect estimate of wTBS.

**Supplementary Table 3: Validation cohort**

| **Variables** | **External cohort (n=162)** |
| --- | --- |
| Tumor size (cm) | 7.1 (4.3-9.9) |
| Tumor nodule number, n (%) |  |
| 1 | 112 (69.1) |
| 2-3 | 23 (14.2) |
| 4-5 | 11 (6.8) |
| >5 | 16 (9.9) |
| Simplified wTBS | 13 (9.6-18.8) |
| Early recurrence, n (%) | 74 (46) |
| No early recurrence, n (%) | 87 (54) |
| Median RFS, months (95% CI) | 9 (5-24) |

Data presented as median and interquartile range, unless otherwise indicated. CI, confidence interval; RFS, recurrence-free survival; wTBS, weighted tumor burden score. Early recurrence status was unavailable for one patient.

**Supplementary Table 4: Model performance and calibration of the wTBS for prediction of early recurrence in the derivation and external validation cohorts.**

| **Cohort** | **AUC (95% CI)** | **Sensitivity** | **Specificity** | **Brier score** | **Calibration intercept** | **Calibration slope** |
| --- | --- | --- | --- | --- | --- | --- |
| Derivation | 0.694 (0.621–0.767) | 61.0 % | 68.0 % | 0.220 | 0.000 | 1.000 |
| External validation | 0.752 (0.677–0.828) | 68.9 % | 75.9 % | 0.210 | -0.057 | 1.053 |

AUC, area under the receiver operating characteristic curve; CI, confidence interval. Sensitivity and specificity were calculated at the predefined wTBS cutoff of ≥14. Calibration performance was assessed using the calibration intercept and calibration slope. Lower Brier scores indicate better overall model performance.

**Supplementary Table 5: Recurrence patterns and treatment modalities among patients with tumor recurrence.**

| **Variables** | **n (%)** |
| --- | --- |
| **Recurrence location** | |
| Local | 87 (59.6) |
| Systemic | 29 (19.9) |
| Local and systemic | 30 (20.5) |
| **Treatment intent** | |
| Curative | 24 (16.4) |
| Palliative | 122 (83.6) |
| **Recurrence treatment*** | |
| Chemotherapy | 101 (69.2) |
| Immunotherapy | 30 (20.5) |
| Repeat surgery | 22 (15.1) |
| Radiotherapy | 20 (13.7) |
| SIRT | 10 (6.8) |
| RFA | 4 (2.7) |
| TACE | 3 (2.1) |
| HIPEC | 1 (0.7) |

*Multiple treatment modalities were possible; therefore, percentages may exceed 100%. HIPEC, hyperthermic intraperitoneal chemotherapy; RFA, radiofrequency ablation; SIRT, selective internal radiation therapy; TACE, transarterial chemoembolization.
